# Hallux valgus deformity and postural sway: a cross-sectional study

**DOI:** 10.1186/s12891-021-04385-4

**Published:** 2021-05-31

**Authors:** Hiroaki Omae, Takashi Ohsawa, Naohiro Hio, Kazuhiko Tsunoda, Takuya Omodaka, Shogo Hashimoto, Akira Ueno, Tsuyoshi Tajika, Yoichi Iizuka, Hirotaka Chikuda

**Affiliations:** 1 Department of Orthopaedic Surgery, Gunma University Graduate School of Medicine, 3-39-22, Showa, Gunma 371-8511 Maebashi, Japan; 2 East Maebashi Orthopaedic Hospital Center of Foot and Ankle Surgery, 1302-2, Nishiomuro, Gunma 379-2104 Maebashi, Japan; 3 Department of Orthopaedic Surgery, Kiryu Orthopaedic Hospital, 284-1, Ainoshima, Hirosawa-machi, Gunma 376-0014 Kiryu, Japan

**Keywords:** Photographic hallux valgus angle, Anteroposterior postural sway, Force plate

## Abstract

**Background:**

Hallux valgus deformity has been reported to be associated with increased postural sway. However, the direction and magnitude of postural sway associated with hallux valgus remain inconclusive. We assessed the association between hallux valgus deformity and postural sway using a force plate.

**Methods:**

The subjects were 169 healthy volunteers, > 40 years old (63 males, 106 females, average age: 66.0 ± 12.4 years old), who took part in an annual medical examination. We investigated the photographic hallux valgus angle (°), total trajectory length of the gravity center fluctuation (mm), area of the center of pressure (mm^2^), mediolateral and anteroposterior postural sway (mm) in a standing position with 2-legged stance and eyes open, hallux pain (Numerical Rating Scale), trunk and lower limb muscle mass (kg). We classified the subjects into a hallux valgus group (*n* = 44, photographic hallux valgus angle of 1 or both feet ≥ 20°) and a no hallux valgus group (*n* = 125, photographic hallux valgus angle of both feet < 20°) and analyzed the relationship between hallux valgus and postural sway.

**Results:**

The anteroposterior postural sway in the hallux valgus group (6.5 ± 2.8) was significantly greater than in the no hallux valgus group (5.4 ± 2.2, *p* = 0.014), and the lower limb muscle mass in the hallux valgus group (12.4 ± 2.2) was significantly smaller than in the no hallux valgus group (13.5 ± 3.2, *p* = 0.016). The total value of the photographic hallux valgus angle on both feet was positively correlated with the anteroposterior postural sway (*p* = 0.021) and negatively correlated with the lower limb muscle mass (*p* = 0.038). The presence of hallux valgus (*p* = 0.024) and photographic hallux valgus angle (*p* = 0.008) were independently related to the magnitude of anteroposterior postural sway.

**Conclusions:**

Hallux valgus deformity and its severity were positively associated with the magnitude of the anteroposterior postural sway.

**Trial registration:**

2017 − 135. Registered 22 August 2017.

## Background

Hallux valgus (HV) is one of the most common foot deformities in adults [1], with a reported prevalence of up to about 30 % [2, 3]. There have been studies showing that HV is associated with greater postural sway, and the presence of HV is assumed to be related to the risk of fall for this reason [4–6]. A study showed that falls from the standing position and with eyes open were associated with not mediolateral postural sway but anteroposterior sway, and the postural sway in fallers was 25 % greater than that in non-fallers [7]. However, the direction and magnitude of postural sway associated with the presence or severity of HV remain inconclusive. Furthermore, previous studies have suggested that muscle mass is a potential confounder of the HV status and postural sway [8–13].

The present study clarified the relationship between the direction and magnitude of postural sway and the presence or severity of HV using a multivariate analysis adjusted for potential confounders, including muscle mass.

## Materials and methods

### Subjects

In a mountain village (Katashina Village, Gunma, Japan, population 4573, 2230 males, 2343 females in 2017), local medical examinations are administered annually to screen for lifestyle-related diseases. The total number of participants in 2017 was 946. Of these, we recruited subjects who were > 40 years old for a foot checkup by board-certified orthopedic surgeons, regardless of the presence of symptoms. Ultimately, 173 healthy volunteers participated in the checkup.

 All of the individuals provided their written informed consent and responded to a baseline questionnaire, which asked for information, such as the age and gender. Of the 173 participants, a total of 169 (63 males, 106 females, average age: 66.0 ± 12.4 years old) who satisfied all of the examination criteria below were included in the present study, which was approved by our institutional review board. All procedures were performed according to the Declaration of Helsinki.

### Measurement of the photographic HV angle (pHVA)

**Fig. 1:**
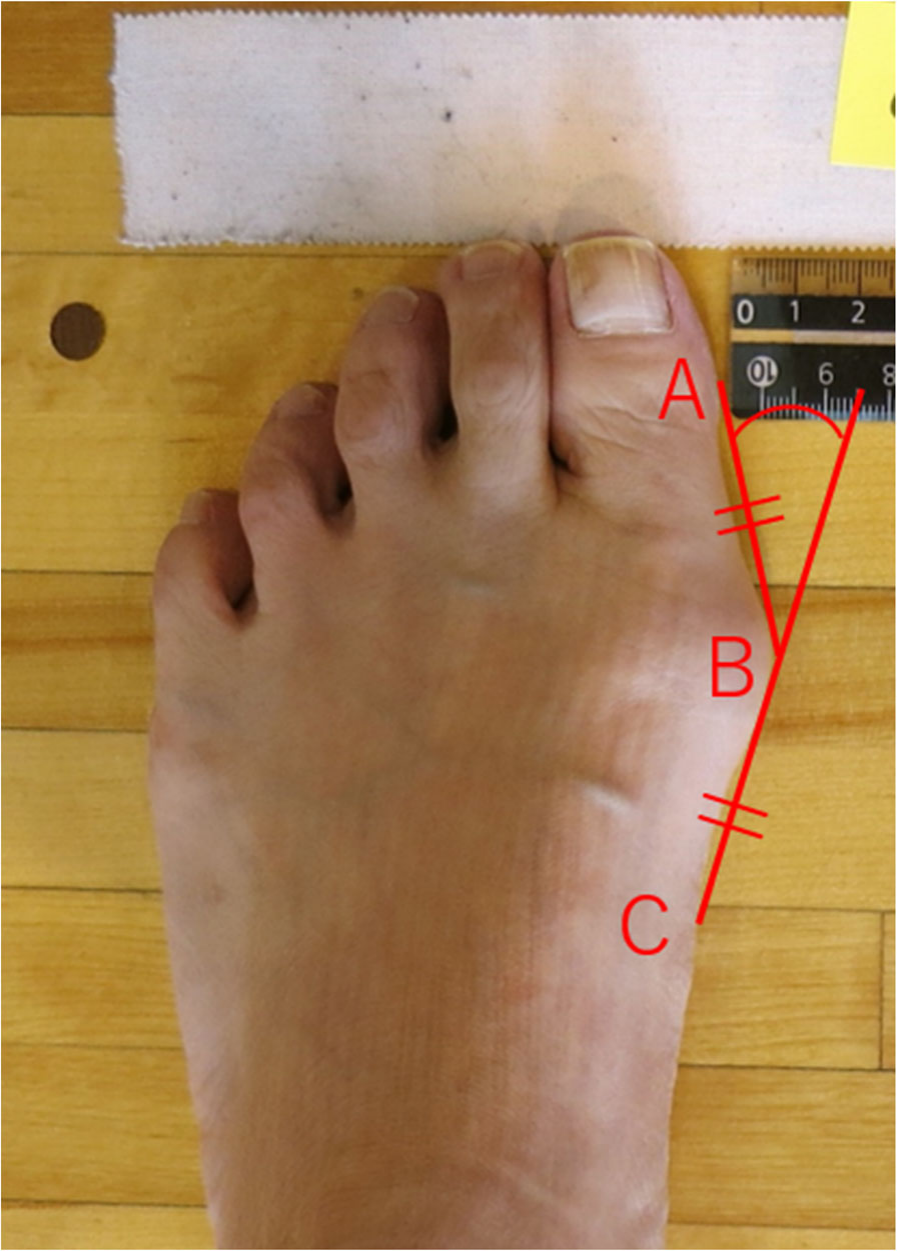
Measurement of the photographic hallux valgus angle. **A**: The inside edge of the hallux. **B**: The inside edge of the head part of the first metatarsal bone. **C**: The inside edge of the part of the first metatarsal bone defined as the length of AB = BC. Photographic hallux valgus angle: The angle formed by AB and BC

With a digital camera, we took photographs of the participants’ feet in the standing position from a 15° inclined angle relative to the vertical direction from a distance of 100 cm and then measured the pHVA (Fig. 1) [14, 15]. First, we drew a tangential line from the inside edge of the hallux (A) to the inside edge of the head part of the first metatarsal bone (B). We then drew a line of the same length as AB from point B in the direction of the heel. Where the line came into contact with the inside edge of the first metatarsal bone was defined as point C. Using an angle measurement application (hakarun®; onegland.net, Shizuoka, Japan), two examiners (HO and KT) measured the pHVA as the angle formed by AB and BC.

A previous study reported that pHVA values were lower than the radiographic HV angle (rHVA) values, with a mean difference of -5.3° (95 % confidence interval [CI], -4.3 to -6.2) [15]. HV is generally defined as an rHVA of ≥ 20° [16]. In the present study, to prevent classifying participants with an rHVA < 20° into the HV group, we set the cut-off as a pHVA of 20° and classified subjects into the HV group (pHVA of 1 or both feet ≥ 20°) and no HV group (pHVA of both feet < 20°). In addition, to analyze the relationship between the severity of HV and the magnitude of postural sway, we calculated the total pHVA for both feet.

### Hallux pain

We asked participants about pain in their right and left hallux or first metatarsal joint while standing on both feet. We assessed the subjective pain of both feet using the Numerical Rating Scale (NRS) (0–10), in which a higher score indicates greater pain, and we further calculated the total NRS score for both feet.

### Postural sway

**Fig. 2:**
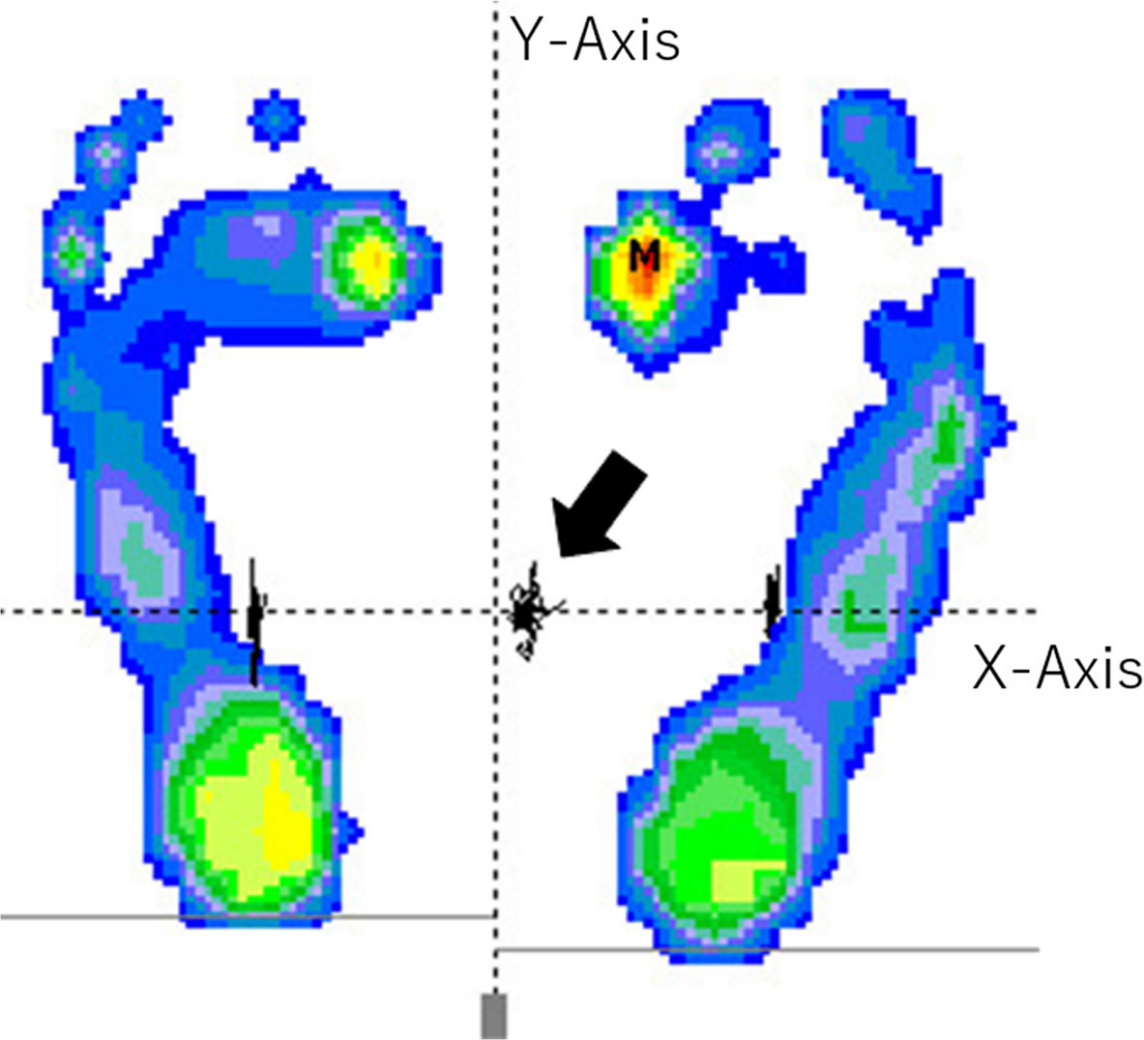
Postural sway measured using a force plate. The trajectory of the center of gravity (black arrow)

Using a force plate (WIN POD®; medicapteurs, Balma, France), we assessed participants’ static balance in standing position with 2-legged stance and eyes open [5, 17] (Fig. 2). We asked participants to stand on the force plate barefoot, with heels 10 cm apart, and to stare at a point 1.5 m away from the force plate for 30 s. From a safety perspective, measurements in a single-leg stance or with the eyes closed were not conducted. We recorded the total trajectory length acquired by the measurement of the gravity center fluctuation (mm), area of the center of pressure (COP; the area of an oval circumscribed to the gravity center fluctuation; mm^2^) and mediolateral and anteroposterior postural sway (mm). The mediolateral axis of the center of gravity was defined as the X-axis, and the anteroposterior axis of the center of gravity was defined as the Y-axis. The magnitude of the mediolateral and anteroposterior postural sway was expressed as the root-mean-square COP displacement, relative to the mean (mm) [7].

### Measurement of the body muscle mass

Using a bioelectrical impedance analysis (Tanita MC-780 A multi frequency segmental body composition analyzer; Tanita Co., Ltd., Tokyo, Japan), we investigated the trunk and lower limb muscle mass [18, 19]. The participants stepped onto the analyzer barefoot, each foot on 2 electrodes, and the examiner entered the participants’ information (age, gender and height). Once the body mass was assessed by the scale, the participants held 2 electrodes gripped in each hand during impedance measurement, which was performed for 20 s. Segmental muscle mass values were indicated on the digital screen (trunk, left and right lower limbs). We then calculated the total muscle mass of both lower limbs (lower limb muscle mass).

### Statistical analyses

We assessed the intraclass correlation coefficients (ICCs_1.1_) and interclass correlation coefficients (ICCs_2.1_) for the procedure used to measure the pHVA. We performed univariate analyses using Welch’s *t*-test and the chi-square test, and compared the HV and no HV groups. Using Spearman’s correlation coefficient, we then analyzed the single correlation between the pHVA and magnitude of postural sway. After adjusting for potential confounders, we used a multiple linear regression model to further confirm the association between the presence of HV or pHVA and the magnitude of postural sway. A p-value of 0.05 was considered statistically significant. All statistical analyses were conducted using a statistical analysis system (IBM SPSS Statistics 26 software package; IBM Japan, Tokyo, Japan).

The sample size was calculated (α = 0.05, β = 0.2) before the present study. The minimum sample size was assessed using a statistical power analysis program (G*Power Version 3.1.; Universität Düsseldorf, Düsseldorf, Germany). The minimum sample size required for each group to achieve a statistical power of > 0.8 was 33 in the HV group and 79 in the no HV group [2, 5].

## Results

Regarding the measurement of the pHVA, the ICCs_1.1_ was 0.987, and the ICCs_2.1_ was 0.983. The HV group had 44 subjects (13 males, 31 females), whereas the no HV group had 125 subjects (50 males, 75 females). According to the univariate analysis, there were no significant differences between the HV and no HV groups with regard to the age, gender, or BMI. The anteroposterior postural sway in the HV group was significantly greater than that in the no HV group, whereas the difference in the mediolateral postural sway between the two groups was not significant. The lower limb muscle mass in the HV group was significantly smaller than that in the no HV group. There were no significant differences between the two groups with regard to the total NRS score for both feet, total trajectory length, area of COP, or trunk muscle mass (Table 1). In a multiple linear regression model, the magnitude of the anteroposterior postural sway was set as dependent variable. In addition to the presence of HV, the age, gender and lower limb muscle mass were set as independent variables, since these variables were assumed to be potential confounders. In this model, only the presence of HV was significantly related to the magnitude of the anteroposterior postural sway (Table 2).

**Table 1:** Results of a univariate analysis of factors related to the presence of hallux valgus

	HV group (*n* = 44)	No HV group (*n* = 125)	*p-value*
Age (years)	69.0 ± 11.6	65.7 ± 11.6	0.108^a^
Gender			
Females	31	75	
Males	13	50	0.217^b^
BMI (kg/m^2^)	22.7 ± 2.9	23.7 ± 3.0	0.058^a^
Total NRS score for both feet	0.1 ± 0.3	0.4 ± 2.0	0.258^a^
Postural sway			
Total trajectory length (mm)	358.6 ± 131.3	328.6 ± 205.5	0.437^a^
Area of COP (mm^2^)	303.9 ± 195.8	238.7 ± 178.1	0.056^a^
The mediolateral postural sway (mm)	4.5 ± 2.0	3.9 ± 1.9	0.090^a^
The anteroposterior postural sway (mm)	6.5 ± 2.8	5.4 ± 2.2	0.014^* a^
Body muscle mass (kg)			
Trunk	22.6 ± 3.8	22.3 ± 3.5	0.620^a^
Lower limbs	12.4 ± 2.2	13.5 ± 3.2	0.016^* a^

The values are given as the mean and standard deviation

*HV* hallux valgus, *BMI* body mass index, *COP* center of pressure, *NRS* numerical rating scale

^a^Welch’s test, ^b^Chi-square test, * *p* < 0.05

**Table 2:** Regression analysis findings of the factors associated with the magnitude of the anteroposterior postural sway

	Standardized partial regression coefficient β	*p-value*
Age (years)	0.128	0.196
Gender	0.092	0.463
The presence of HV	0.176	0.024^*^
The lower limb muscle mass (kg)	0.073	0.584

The dependent variable was the magnitude of the anteroposterior postural sway. The independent variables were the age, gender, presence of HV and lower limb muscle mass

*HV* hallux valgus

*p < 0.05

**Fig. 3:**
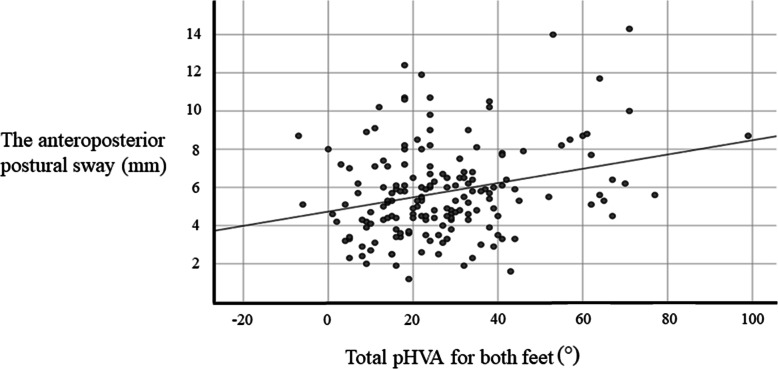
Correlation between the severity of hallux valgus and the anteroposterior postural sway. The total value of the photographic hallux valgus angle for both feet was positively correlated with the anteroposterior postural sway

The Spearman’s correlation analysis showed that the total value of pHVA on both feet was positively correlated with the anteroposterior postural sway and negatively correlated with the lower limb muscle mass (Fig. 3; Table 3). In a multiple linear regression model with the anteroposterior postural sway as the dependent variable and the total value of the pHVA on both feet, age, gender and lower limb muscle mass as independent variables, only the total pHVA for both feet was independently correlated with the magnitude of the anteroposterior postural sway (Table 4).

**Table 3:** Correlations between the severity of hallux valgus and measured variables

	Total pHVA for both feet (°)	
	*r*	*P*
Age (years)	0.120	0.119
BMI (kg/m²)	-0.128	0.096
The anteroposterior postural sway (mm)	0.177^*^	0.021
The lower limb muscle mass (kg)	-0.160^*^	0.038

Data are shown as Spearman’s correlation coefficient (*r*)

*BMI* body mass index, *NRS* numerical rating scale, *pHVA* photographic hallux valgus angle

*p < 0.05

**Table 4:** Regression analysis findings of the factors associated with the magnitude of the anteroposterior postural sway

	Standardized partial regression coefficient *β*	*p-value*
Age (years)	0.102	0.335
Gender	0.044	0.757
Total pHVA for both feet (°)	0.214	0.008^*^
The lower limb muscle mass (kg)	0.033	0.837

The dependent variable was the magnitude of the anteroposterior postural sway. The independent variables were the age, gender, total value of pHVA and lower limb muscle mass

*pHVA* photographic hallux valgus angle

*p < 0.05

## Discussion

The present study demonstrated two main findings. First, the presence of HV was independently related to the magnitude of the anteroposterior postural sway. Second, the severity of HV deformity was also related to the magnitude of the anteroposterior postural sway.

Previous studies have shown that HV is inversely related to the hallux plantar load pressure [8, 20], hallux plantarflexion strength [5] and tactile sensation or proprioception [21], all of which are important for preventing postural sway while the gravity center moves to the anterior part of the foot. These reasons may explain the potential relationship between HV deformity and postural sway in the anteroposterior direction. In line with our findings, a study also reported that the presence of HV was significantly related to the postural sway, although the sway was found in the mediolateral direction [6]. In that previous study, the subjects were > 20 years old, HV was defined as an rHVA > 15° on radiographs, and the measurement time was 70 s. Thus, such subjects and basic procedures in the previous study might have resulted in the direction of postural sway differing from the present findings.

Several researchers reported that HV was an important risk factor for falls [22–24]. In addition, a study found that, in the standing position with a 2-legged stance and eyes open, fallers tended to have a greater anteroposterior postural sway (an increase of 25 %) than non-fallers [7]. In line with that study, we found that the anteroposterior postural sway in the HV group was increased by 20 % compared to that in the no HV group. Although further studies are needed, based on the present as well as previous reports, falls in subjects with HV may be related to the anteroposterior postural sway. Furthermore, HV deformity can progress over time [25], and the severity of HV was correlated with the magnitude of the postural sway in the present study. Thus, it may be important to monitor the progress of a patient’s HV deformity.

Several limitations associated with the present study warrant mention. First, we did not investigate the history of fall in this study. Therefore, we could not conclude that increased postural sway was directly related to fall. Second, measurements in a single-leg stance and eyes closed were not conducted from the perspective of safety. Finally, ocular disease might have affected the subjects’ ability to stare at a point 1.5 m from the force plate during the measurement of postural sway. Despite these limitations, a strength of the present study is that we clarified for the first time the association of the presence and severity of HV deformity and the anteroposterior postural sway among healthy volunteers.

## Conclusions

The presence and severity of HV deformity were independent factors related to the anteroposterior postural sway in a standing position with a 2-legged stance and eyes open.

## Data Availability

The data are available from the corresponding author upon reasonable request.

## Abbreviations

BMI: Body mass index
COP: Center of pressure
HV: Hallux valgus
ICC: Intraclass correlation coefficients
NRS: Numerical rating scale
pHVA: Photographic hallux valgus angle
